# Application of Bi_12_ZnO_20_ Sillenite as an Efficient Photocatalyst for Wastewater Treatment: Removal of Both Organic and Inorganic Compounds

**DOI:** 10.3390/ma14185409

**Published:** 2021-09-18

**Authors:** Oussama Baaloudj, Noureddine Nasrallah, Hamza Kenfoud, Faisal Algethami, Abueliz Modwi, Ahlem Guesmi, Aymen Amine Assadi, Lotfi Khezami

**Affiliations:** 1 Laboratory of Reaction Engineering, Faculty of Mechanical Engineering and Process Engineering, University of Science and Technology Houari Boumediene (USTHB), BP 32, Algiers 16111, Algeria; obaaloudj@gmail.com (O.B.); nas_nour@yahoo.fr (N.N.); hamza.kenfoud.93@gmail.com (H.K.); 2 Department of Chemistry, College of Sciences, Imam Mohammad Ibn Saud Islamic University, P.O. Box 5701, Riyadh 11432, Saudi Arabia; falgethami@imamu.edu.sa (F.A.); amalkasme@imamu.edu.sa (A.G.); 3 Department of Chemistry, College of Science and Arts, Qassim University, Ar Rass 51921, Saudi Arabia; abuelizkh81@gmail.com; 4 CNRS, Ecole Nationale Supérieure de Chimie de Rennes, Univ. Rennes, ISCR-UMR 6226, F-35000 Rennes, France

**Keywords:** sillenite, Rietveld, optical bandgap, photodegradation, photoreduction

## Abstract

This work aims to synthesize and characterize a material that can be used as an effective catalyst for photocatalytic application to remove both organic and inorganic compounds from wastewater. In this context, sillenite Bi_12_ZnO_20_ (BZO) in a pure phase was synthesized using the sol–gel method. Before calcination, differential scanning calorimetry (DSC) analysis was done to determine the temperature of the formation of the sillenite phase, which was found to be 800 °C. After calcination, the phase was identified by X-ray diffraction (XRD) and then refined using the Rietveld refinement technique. The results prove that BZO crystals have a cubic symmetry with the space group I23 (N°197); the lattice parameters of the structure were also determined. From the crystalline size, the surface area was estimated using the Brunauer-Emmett-Teller (BET) method, which was found to be 11.22 m^2^/g. The formation of sillenite was also checked using the Raman technique. The morphology of the crystals was visualized using electron scanning microscope (SEM) analysis. After that, the optical properties of BZO were investigated by diffuse reflectance spectroscopy (DRS) and photoluminescence (PL); an optical gap of 2.9 eV was found. In the final step, the photocatalytic activity of the BZO crystals was evaluated for the removal of inorganic and organic pollutants, namely hexavalent chromium Cr(VI) and Cefixime (CFX). An efficient removal rate was achieved for both contaminants within only 3 h, with a 94.34% degradation rate for CFX and a 77.19% reduction rate for Cr(VI). Additionally, a kinetic study was carried out using a first-order model, and the results showed that the kinetic properties are compatible with this model. According to these findings, we can conclude that the sillenite BZO can be used as an efficient photocatalyst for wastewater treatment by eliminating both organic and inorganic compounds.

## 1. Introduction

Water pollution is a significant concern that harms the environment, life, and living creatures [1,2], and has been one of the main worldwide concerns in terms of environmental remediation over the last few decades [3]. Organic contaminants and inorganic heavy metals are the most common substances that can cause harmful water pollution [4,5,6,7]. No matter how small the concentration of these pollutants in water may be, the organs of living beings might be damaged by long-term exposure to them [8,9]. Companies, such as pharmaceutical, textile, and thinner and metallurgical industries, are the primary sources of these water pollutants [10]. Among these compounds, antibiotics as organic compounds and heavy metals (HMs) as inorganic compounds are two of the most hazardous contaminants.

Starting with antibiotics, which are a topical example of organic pollutants and are extremely dangerous due to their wide use in both human and veterinary drugs [11], an analysis of antibiotic use has shown that global antibiotic use was recorded at approximately 200,000 tons and has grown by 65% over only 15 years from 2000 and will double by around 2030 [12,13]. This widespread use assures the presence of these antibiotics in different water supplies, in quantities extending from ng/L to µg/L [14]. Taking cefixime (CFX) as an example, it has been detected in various water environments with quantities ranging from 278 to 422 ng/L [15,16]. However, even in small antibiotics doses, antibiotic bacterial resistance (ABR) can increase and develop. This type of bacteria is well-known as a severe threat to world health in the 21st century, because they cause nearly a million deaths worldwide each year [17,18,19,20]. For this reason, we selected CFX as an organic pollutant for this study.

Along with these organic pollutants, heavy metals (HMs) as inorganic contaminants are also toxic to living beings above a threshold value because they are absorbed, kept, and concentrated by many life forms [21]. Unlike organic contaminants that are sometimes biodegraded in water, heavy metals can not be wholly removed from water, and are reduced to other metal ions that are less damaging [22]. Amidst the rapid growth of industry and human activities, water is constantly polluted by large amounts of heavy metal ions, such as Cr (VI), Pb (II), O (III), Hg (II), and Cu (II) [8]. Among these HMs, chromium is a highly toxic metal, widely found in many industrial processes, such as leather tanning, metal plating, manufacturing of dye, stereotyping, ink, and paint and paper production [23,24]. It exists in water environments in various oxidation states, mainly hexavalent Cr(VI) and trivalent Cr(III). Cr(VI) is the most stable state and is more harmful to humans than other states, such as Cr(III, IV and V), because it is carcinogenic, mutagenic, and has high mobility characteristics [1,22]. For these reasons, the World Health Organization (WHO) has limited Cr(VI) concentrations in all water sources to below 0.05 mg [25]. These ions are formed mainly from chromate ions, CrO_4_^2−^, and dichromate ions, Cr_2_O_7_^2−^. However, as mentioned previously, metal ions can not be totally eradicated from water, but the ideal elimination is to convert Cr(VI) ions to less damaging Cr(III) ions [5].

In this respect, for the elimination of both inorganic and organic pollutants, photocatalysis was proposed as it has shown promising results for the elimination of a large number of pollutants. It has been a widely studied technology since the 1970s due to its advantages, such as its low cost, ecofriendliness, and reusability. The photocatalysis process generally depends on a photocatalyst and an excitation source, such as light. When the excitation source is exposed to a photocatalyst, electrons e^−^ and holes h^+^ are produced, which can eliminate organic and inorganic compounds via oxidation and reduction reactions [26]. Semiconductors are the most common catalyst in photocatalytic applications, as they are efficient and cost-effective. Bismuth-based semiconductors have attracted considerable research interest because bismuth ions are favorite materials for catalysts [27]. Furthermore, as bismuth has toxic ions when they are free, synthesizing a stable useful material can help to reduce their risk in the environment [28]. Within the bismuth semiconductor category, sillenite forms of Bi_12_[M]O_20_ have attracted many scientists because of their unique crystal structures, interesting photochromic qualities, peculiar electronics, and dielectric photorefractive properties, promising optical activity [29,30,31]. There are a large number of new sillenites that have been used as photocatalysts in previous research, such as Bi_12_TiO_20_ [32], Bi_12_GeO_20_ [33], Bi_12_NiO_19_ [34], Bi_12_CoO_20_ [35], and Bi_12_ZnO_20_ [36]. Among these, the bismuth zincate sillenite Bi_12_ZnO_20_ (BZO) has drawn tremendous interest because of its tiny band gap, its stability, and its high photoconductivity [37,38,39]. BZO is an environmentally friendly semiconductor without lead, which makes its application very promising.

This work aimed to synthesize and use a new photocatalyst with an interesting photocatalytic activity to remove various types of pollutants. Cefixime (CFX) was selected as an organic pollutant and hexavalent chromium Cr(VI) as an inorganic contaminant for application in the study. The efficient catalyst used in this research was the sillenite Bi_12_ZnO_20_. It was successfully synthesized in a pure form using the sol–gel method. The obtained phase was characterized and identified using various techniques, including X-ray diffraction (XRD), Raman, SEM, DRS, and photoluminescence. Following catalyst identification, removal tests for the chosen pollutants CFX and Cr(VI) under optimal photocatalytic conditions were conducted. To our knowledge, only one study has dealt with the synthesis and use of BZO as a photocatalyst for the removal of antibiotics. Moreover, no study has been performed yet concerning the reduction of organic pollutants using this catalyst. This work focuses on experimental and theoretical investigations of the structure, and optical and photocatalytic properties of this interesting catalyst, which is in line with the scope of the Materials journal.

## 2. Materials and Methods

### 2.1. Chemicals

The following products were used in this investigation: bismuth nitrate pentahydrate [Bi(NO_3_)_3_∙5H_2_O] from Chem-Lab (Zedelgem, Belgium) with a purity of 98.5% and zinc nitrate hexahydrate [Zn(NO_3_)_2_∙6H_2_O] from Biochem (Barcelona, Espagne) with a purity of 98%; cefixime trihydrate C_16_H_15_N_5_O_7_S_2_ and polyvinylpyrrolidone (PVP K30) were supplied by a pharmaceutical company (Algiers, Algeria), Pharmalliance; ethanol and nitric acid (HNO_3_) were provided by Sigma-Aldrich (Saint-Quentin-Fallavier, France). Potassium dichromate, K_2_Cr_2_O_7_, was supplied by Emsure (Algiers, Algeria). All compounds were utilized as purchased without further purification. All preparations were made using distilled water.

### 2.2. Synthesis of Bi_12_ZnO_20_ Crystals

**Figure 1:**
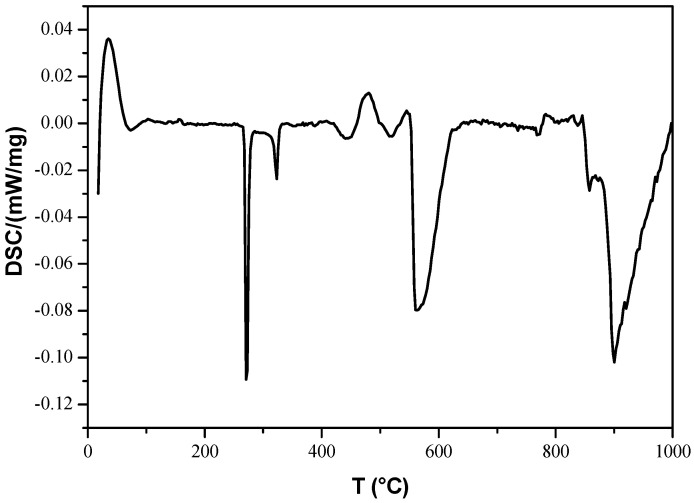
The DSC curve of the Bi_12_ZnO_20_ nanoparticles.

Bi_12_ZnO_20_ (ZBO) was synthesized using the PVP sol–gel method. The gel was prepared using a solution with a 15% *w*/*w* concentration of PVP K30 (to obtain the complexing role [40]). [N_2_O_6_Zn 6H_2_O] and [Bi(NO_3_)_3_∙5H_2_O] with ratio amounts (1:12) were dissolved in ethanol with an excess of 5% nitric acid to ensure that the nitrates were soluble. After a total solubility, the obtained solution was mixed with the gel and then placed on a hot plate at 50 °C for 1 h. The obtained gel was evaporated at 80 °C for 24 h and then dried at 200 °C for 6 h. A precursor powder, called Xerogel, was obtained after drying using an auto combustion reaction. The obtained Xerogel was crushed in an agate mortar and then calcined at 800 °C (which was selected based on DSC analysis, Figure 1) for 6 h in an air oven in a programmed furnace (5 °C min^−1^). Six hours was selected as the calcination time because it was found to be the maximum calcination temperature; calcining the sample for longer than 6 h can cause it to melt and stick in the oven. Moreover, the highest calcination time was chosen to improve the crystallinity, as well as to eliminate carbonaceous residue left from the combustion reaction [13,36]. The obtained whitish-yellow powder was subjected to phase identification, optical characterization, and photocatalytic studies.

### 2.3. Catalyst Characterization

XRD analysis was performed using a Phillips PW 1730 (Eindhoven, The Netherlands) with 2θ ranging from 5 to 80°. The lattice parameters of the structure were refined using the Rietveld method using the software, MAUD (version 2.93). Structural presentation was realized using the Vesta program. Raman spectra were obtained from 100 to 800 cm^−1^ using a Horiba Jobin-Yvon (LabRAM HR, Longjumeau, France). The morphology of the sample was visualized by field emission scanning electron microscopy (FESEM) (JEOL JSM-7610plus, Tokyo, Japan). The sample’s UV–visible diffuse reflectance spectrum (DRS) was obtained from 200 to 800 cm^−1^ using a Cary 5000 UV−vis (Agilent, Stevens Creek Blvd, Santa Clara, CA, USA). The photoluminescence spectra were determined using an FL3-DFX-IHR320 spectrophotometer (Horiba, Longjumeau, France) with a wavelength excitation of 342 nm.

### 2.4. Photocatalytic Performance Tests

The photocatalytic activity of the catalyst was examined for photodegradation of CFX and photoreduction of Cr(VI) in a stirred double-walled reactor. For CFX degradation, the test was carried out by mixing a quantity of BZO (0.1 g) in a 100-mL solution with an initial CFX concentration of 10 mg/L and a neutral pH for an irradiation time of 180 min, which were found to be the optimal conditions. Before exposing the reactor to irradiation, the solution was stirred under dark conditions for 120 min to reach adsorption/desorption equilibrium. The irradiation source was a UVA 24 W lamp (Philips, Eindhoven, The Netherlands) with a UV intensity of 20 mW/cm^2^, placed vertically at 10 cm from the solution surface. The solution temperature was held constant at 25 °C in all photocatalytic applications by a thermostatic bath. During the process of the photocatalysis after illumination, aliquots of 3.0 mL were collected and centrifuged to separate the photocatalyst from the solution, and then measured using a UV-visible spectrophotometer (OPTIZEN, UV-3220UV, Daejeon, Republic of Korea) at λ_max_ = 288 nm, to determine the leftover CFX concentration. The degradation rate of CFX was calculated using Equation (1).

(1) $$\mathrm{Degradation}\;\mathrm{rate}\;\%\;=\frac{\left(C_{\mathrm{ad}}-C\right)}{C_{\mathrm{ad}}}\times 100$$

where *C*_ad_ is the initial concentration after adsorption at (t = 0) and *C* is the concentration after illumination at time (t).

For Cr(VI) reduction, a potassium dichromate solution (K_2_Cr_2_O_7_) was taken at a concentration of 15 mg/L and the same approach as the experiment was used for organic degradation. Oxalic acid as a reduction agent was added to the Cr(VI) to slow down the pair (e−/h+) recombination rate and to prevent the photo corrosion of the catalyst [25]. Cr(VI) removal was followed by measuring the absorption at wavelength 344 nm using a UV-visible spectrophotometer. The reduction rate of Cr(VI) was calculated using Equation (2).

(2) $$\mathrm{Reduction}\;\mathrm{rate}\;\%=\frac{\left(abs_{\mathrm{ad}}-abs\right)}{abs_{\mathrm{ad}}}\times 100$$

## 3. Results

### 3.1. Catalyst Characterization

#### 3.1.1. Investigation of Calcination Temperature

To clarify the calcination temperature effect on the thermal decomposition process and the thermal properties of the Bi_12_ZnO_20_ nanomaterials, the obtained dried gel was examined using DSC analysis at (Mettler Toledo, Bordeaux, France) a heating rate of 5 °C/min. The obtained results are illustrated in Figure 1. It was observed that the Zn-Bi-O gel precursor exhibited two endothermic peaks; the first, an intense peak at ~279 °C, and the second, a small peak at ~324 °C. These two endothermic peaks have been associated with the decomposition of organic matter, including water, and denitrification. At around 510 °C, the reaction became exothermic and the peak at ~550 °C can be attributed to the composition of Bi_2_O_3_ [41]. In the range of 750 to 850 °C, there was another increase, which means an exothermic reaction occurs, which shows the formation of ZBO sillenite. The distortion that occurs after 850 °C was attributed to the sillenite melting, it is well-known that sillenites melt at these temperatures. The optimal calcination temperature can therefore be concluded as 800 °C.

#### 3.1.2. Phase Identification and Structural Investigation

**Figure 2:**
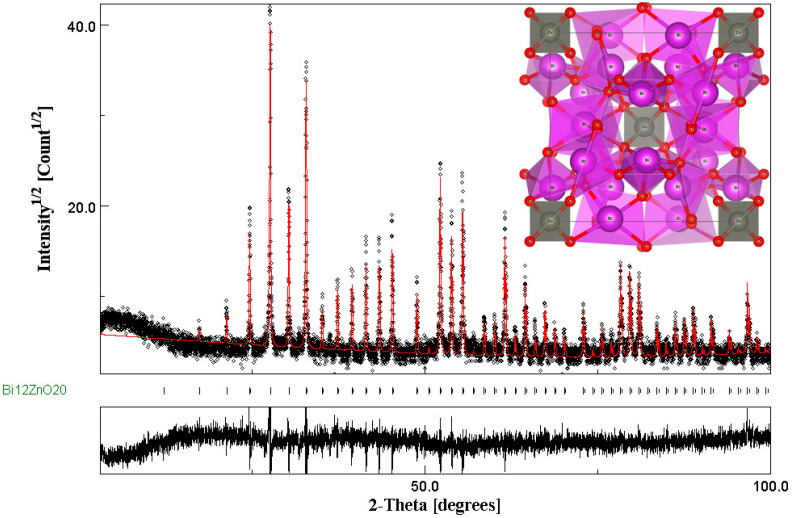
Rietveld refined XRD pattern for the Bi_12_ZnO_20_ nanoparticles. The circles represent experimental points and the red line represents Rietveld refined data. Inset: supercell model for bulk Bi_12_ZnO_20_.

After calcination, the prepared powder was subjected to XRD investigation (Figure 2) to confirm the obtaining of the sillenite phase. The observed diffraction peaks correspond to standard patterns of a cubic bismuth zinc sillenite (PDF #78-1325) [37]. Nonetheless, no peaks corresponding to the precursor Bi_2_O_3_, ZnO, or other impurities were observed, indicating high phase purity. The sharp intensity of the main diffraction peak in the (311) plane exhibits good crystallization of the selenite, which has a monophasic cubic structure with a space group of I23. As is well known, direct measurement of the lattice parameters and determining the lattice of phase structure from XRD patterns is difficult, so the Rietveld refinement method was used to refine them [42].

In this part, the XRD pattern was refined employing the Rietveld method with the help of MAUD software using the I23 space group. The fitting after refinement is presented in Figure 2. The observed and calculated profiles are suitable for one another, and all experimental peaks are approved Bragg 2θ for the space group I23. Using Rietveld after refinement, both the structural and lattice parameters were calculated, such as the lattice constants, atomic positions, the Sig (goodness of fit), and also various R factors, such as R_wp_ (weighted profile factor), R_exp_ (expected weighted profile factor), and R_b_ (Bragg factor). The obtained results were gathered in Table 1. The goodness of fit, Sig, evaluated the fitting quality of the experimental data; the closer to 1, the more the fitting was good; in our case, it was 1.76, which is very significant. This showed the excellent quality of our sillenite phase.

The lattice table results enabled us to construct our exact sillenite structure using Vista software and the resulting structure is shown in Figure 2 (inset), representing a total of 66 atoms in a supercell. The red, purple spheres, and green represent O, Bi, and Zn atoms, respectively.

Compared to our previous study that dealt with the synthesis of BZO using co-precipitation [36], the purity of the catalyst that was synthesized using the sol–gel method was higher than the co-precipitation method. This could be confirmed by taking the Sig (goodness of fit) into account as sol–gel was closer to 1 (around 1.75) and was 3.21 for co-precipitation, which is significantly high.

The average crystallite size of the BZO selenite crystals (59.46 nm) was determined using Scherrer’s equation.

(3) $$D=\frac{K\lambda }{\beta \cos\left(\theta \right)}$$

where *D* is the crystal size, ω is the peak width of half maximum intensity, θ is the diffraction angle, and λ represents the wavelength of the X-ray.

**Figure 3:**
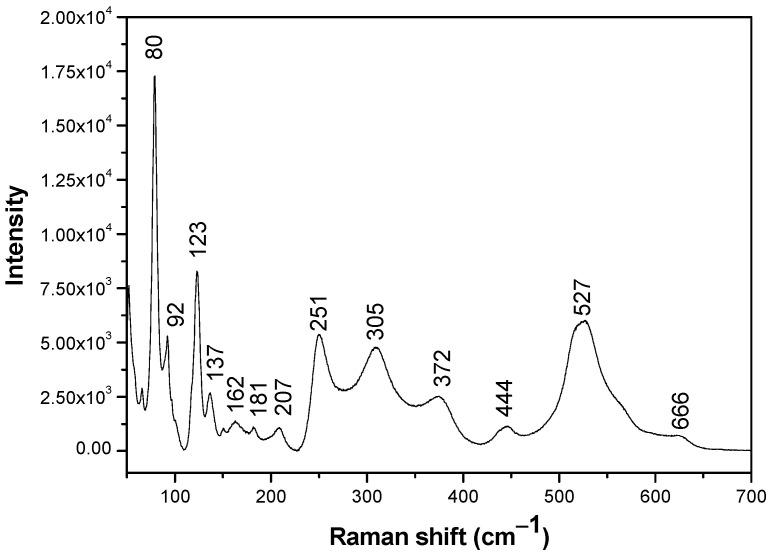
Raman spectra of Bi_12_ZnO_20_.

The photocatalytic process takes place on the photocatalyst’s surface, suggesting that surface area is a crucial parameter that can affect catalyst photocatalytic activity [43]. The greater the photocatalyst’s surface area, the greater the number of convenient reaction sites, which heightens the catalyst activity. Crystallite size is another significant parameter that can influence photocatalytic activity because it correlates well with surface area, where surface area is inversely proportional to crystallite size [44]. Accordingly, the smaller the crystallite size, the greater the surface area available to the catalyst, which also enhances catalyst activity [45]. Therefore, it is very important to determine the surface area of a catalyst. This surface can be determined from crystallite size values by assuming nanocrystalline spheres and using the Brunauer-Emmett-Teller (BET) relation [46].

(4) $$S_{\mathrm{BET}}=\frac{6\times 10^{3}}{D\times \rho }$$

where ρ = 8.91 g/cm^3^ is the density and obtained from the equation, $\rho =\frac{ZM}{N_{A}\;V}$ [10], where *Z* = 2 [35] is the number of model units in a single cell. According to these calculations, the surface area for the sillenite nano-powder is 11.22 m^2^/g. Compared to a previous study with a sample that was synthesized using the co-precipitation method, the special surface area of the sol-gel method (11.22 m^2^/g) was lower than that of the co-precipitation method (12.31 m^2^/g), due mainly to the crystallite size and also to the purity of the phase, as ZnO as an impurity can increase the surface area in the co-precipitation method. Sillenites are high in scatter yield and Raman spectra is a reliable technique that can characterize these sillenite materials [38]. The Raman spectra of BZO crystals are given in Figure 3. As can be seen, there are 12 peaks, from 50 to 700 cm^−1^, these peaks are lattice modes and match pretty well with the form of sillenite [38,47]. Modes detected in 80, 92, 123, and 137 cm^−1^ are due to the vibration and respiration of the Bi and O bands at various positions, wherein modes 162 between 207 cm^−1^ are attributed to the variations of the bands Bi–O–Bi [48]. All of these bismuth modes correspond to the polyhedral BiO_7_ shape. The tiny Raman modes at 251, 305, 372 and 527 cm^−1^ probably correspond to vibrations and breathing of oxygen atoms (O). The two weakest modes at 444 and 666 cm^−1^ are related to the stretching and vibrations of the bonds in the ZnO_4_ tetrahedra.

#### 3.1.3. Morphology Investigation

**Figure 4:**
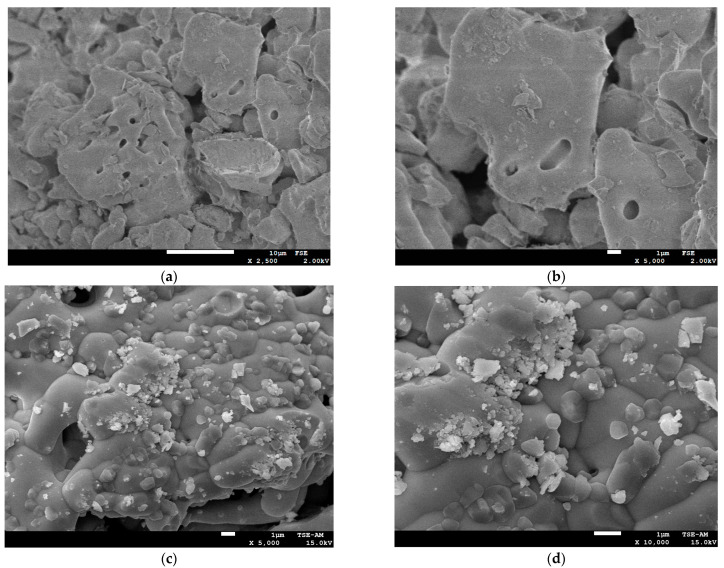
(**a**,**b**) FSE (**c**,**d**) TSE-AM SEM images for the Bi_12_ZnO_20_ crystals.

The surface morphology of the sillenite Bi_12_ZnO_20_ was examined using scanning electron microscopy and is illustrated in Figure 4. The SEM analysis was performed using two different modes, FSE and TSE-AM. The FSE SEM images (a, b) display a clear porous structure that is spherical, and the content of the powder presents large agglomerate particles that are less uniform with large and small compact grains. Furthermore, the formation of nano-crystal grains is observed; the fine nature of the BZO particles is responsible for the agglomeration. The nanocrystals are nearly connected and produce large surface areas that predict enhanced photocatalytic activity [49]. The TSE-AM SEM images (c, d) can support the fact that we obtained nanoparticles since we cannot see unique particles (not agglomeration) larger than 1 μm in the SEM images.

#### 3.1.4. Optical Property Investigation

Information about the efficient charge transfer and the electron-richness in the system of photocatalyst nanoparticles could be extracted from the photoluminescence characteristics [50]. The Bi_12_ZnO_20_ nanocrystal photoluminescence was determined to explore the optical properties obtained after milling and subsequent annealing at 800 °C. The emission spectra were measured using λ = 342 nm as the excitation wavelength.

**Figure 5:**
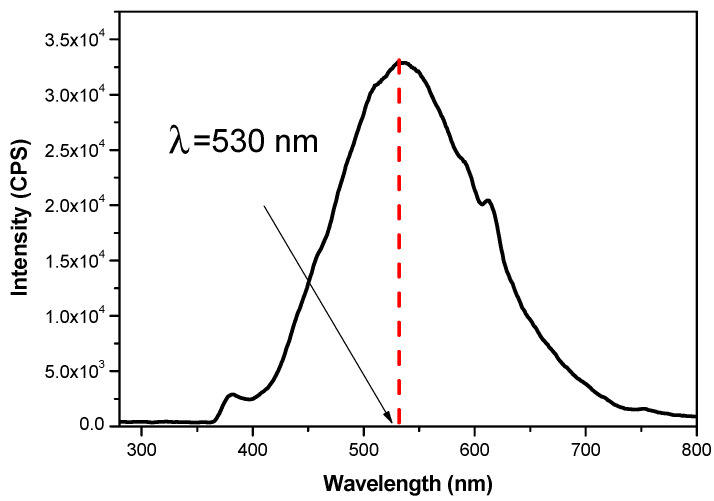
Room temperature PL spectrum for Bi_12_ZnO_20_ nanoparticles.

As shown in Figure 5, at this wavelength, the emission peak for Bi_12_ZnO_20_ nanoparticles has a comparatively weaker intensity than that of pure Bi_2_O_3_, which has been studied before [51]. The peak magnitude at 530 nm was more potent due to photosensitization of the sample and this means a longer electronic life. This also shows that the crystals have a low recombination rate for the photogenerated electron–hole pairs (e^−^/h^+^), confirming that the sillenite phase structure has a slow separation of the photo-excited electron–hole pairs with a low capability of transfer and longer hole diffusion distance [52]. Hence, this helps to greatly enhance photocatalytic activity.

**Figure 6:**
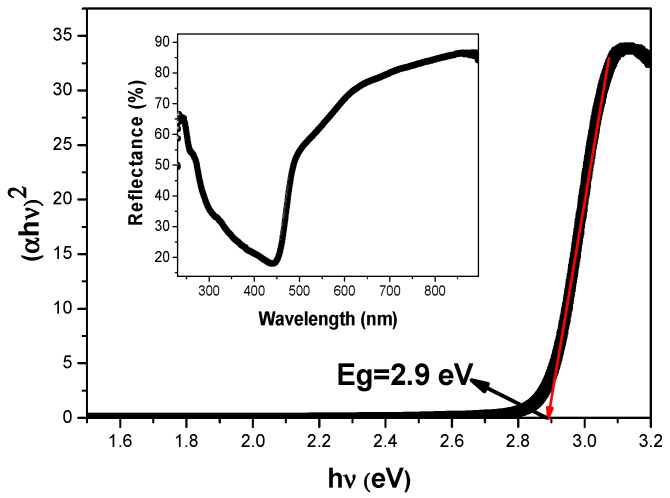
(αhν)^2^ versus photon energy. Inset: UV-vis spectrum of Bi_12_ZnO_20_.

UV-visible diffuse reflectance spectroscopy (DRS) studies play an essential role in the estimation of the optical band gap energy (E_g_) and electronic structures of the metal oxide semiconductor materials [53,54]. To assess the light absorption of our sillenite Bi_12_ZnO_20_ nanopowder, diffuse reflectance spectra were investigated in the range of 200–800 nm at room temperature, as are shown in Figure 6 (inset). From this figure, we see that the sharpness and significant value of the absorption edge at ~475 nm correspond to the band-to-band transition of the sillenite [55]. The diffuse reflectance is transferred to the equivalent absorption coefficient by the Kubelka–Munk function to determine the bandgap energy (E_g_) using the Tauc relation [56].

(5) $$(\alpha h\nu {)}^{\frac{2}{n}}=K\left(h\nu -E_{g}\right)$$

Figure 6 represents (αhν)^2^ versus hν plots of absorption spectra, where α is the absorption and the exponent n is related to the type of optical transmission caused by the absorption of photons, where it is equal to 1 or 4 for direct or indirect transmission, respectively, for the semiconductor. The sillenite Bi_12_ZnO_20_ optical bandgap was determined by fitting a straight line to the linear portion of the curve where the Eg energy is the intercept of the line with the hν axis. The obtained band gap value with a direct optical transition is 2.9 eV. This result shows that the optical properties of our semiconductors, for the photocatalytic reaction, are in the UV light region. For this, a UVA lamp was used in the experiments with the catalyst.

### 3.2. Photocatalytic Performance Applications

Two types of pollutants, cefixime as an organic and hexavalent chromium Cr(VI) as an inorganic pollutant, were proposed to test the photocatalytic activity of BZO crystals. Before starting the experiments, the effect of photolysis was neglected by exposing both pollutants to light without the BZO catalyst, where there was no significant change in their concentrations. Then, in the first part of the experiment, BZO was added to the solution and stirred for 120 min under dark conditions to eliminate the adsorption effect. The BZO crystals adsorbed only a small amount of both pollutants.

#### 3.2.1. Degradation of Organic Pollutant

**Figure 7:**
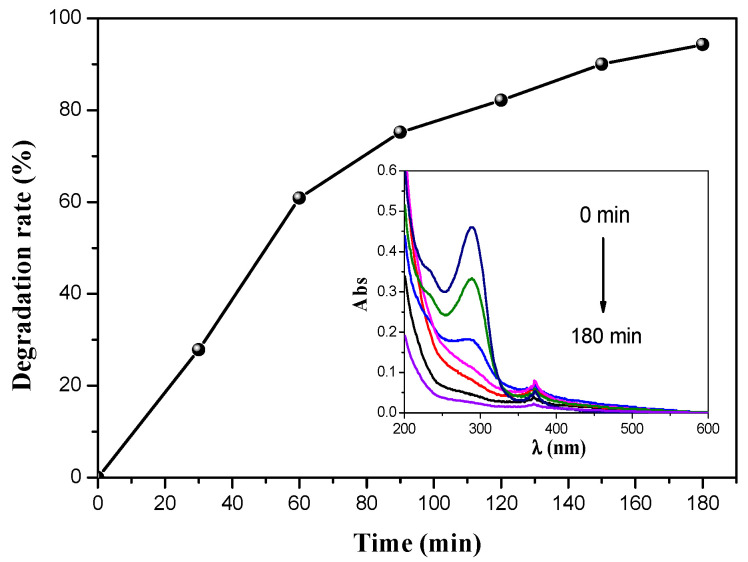
Degradation efficiency on CFX as a function of time; [CFX]_0_ = 10 mg/L, catalyst dose 1 g/L; inset: UV–visible spectra.

The BZO catalyst degradation efficiency was first tested for the degradation of cefixime. The degradation was conducted by applying light to the solution after the elimination of the adsorption effect. The degradation efficiency as a function of time is presented in Figure 7, where the inset figure shows the evolution of the UV-Visible spectra for CFX degradation. As can be seen, the achieved rate of CFX degradation was 94.34% within 3 h, which demonstrated that CFX was successfully removed using BZO crystals as a photocatalyst. This degradation rate was higher than that obtained using others catalysts during our previous studies [13,14]. As BZO crystals have shown great degradation for CFX, it can be concluded that they can be effective catalysts for the removal of organic pollutants.

#### 3.2.2. Reduction of Inorganic Pollutant

**Figure 8:**
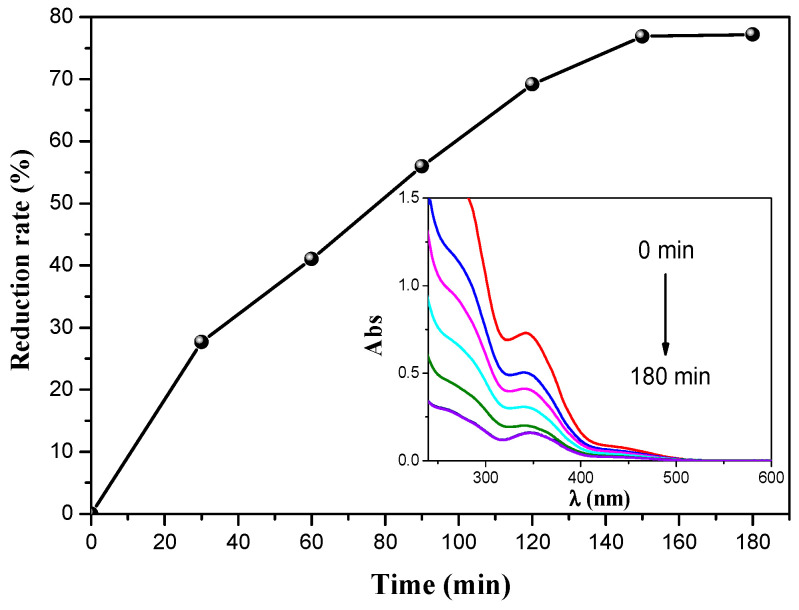
Reduction efficiency on Cr(VI) as a function of time; [Cr]_0_ = 30 mg/L, catalyst dose 1 g/L; inset: UV–visible spectra.

Following this promising result concerning removing organic pollutants, the catalyst was tested to reduce an inorganic pollutant, Cr(VI). The experiment was conducted using a BZO catalyst, maintaining all conditions similar to those of the degradation experiment. The experiment was performed by adding 0.1 g of BZO catalyst in 100 mL of K_2_Cr_2_O_7_ solution (15 mg/L) at pH ~6. The photoreduction of Cr(VI) as a function of time is shown in Figure 8, where the inset figure presents the evolution of the UV-visible spectra for Cr(VI) reduction. The results show that the photocatalytic efficiency in Cr(VI) reduction achieved 77.19% removal within 180 min of irradiation time. This removal was accomplished by reducing Cr_2_O_7_^2−^ to Cr^3+^, as indicated by the equation below [1].

(6) $${\mathrm{Cr}}_{2}O_{7}{}^{-2}+14{\;H}^{+}+6{\;e}^{-}\to 2{\;\mathrm{Cr}}^{+3}+7{\;H}_{2}O$$

As observed, the catalyst has an efficient performance in the removal of both types of pollutants of 94.34% CFX degradation and 77.19% Cr(VI) reduction within only 3 h. This finding allows it to be used as an efficient catalyst to treat organic and inorganic compounds in industrial wastewater.

### 3.3. Kinetic Study of the Photodegradation and Photoreduction

The kinetics of the photodegradation of organic and the photoreduction of inorganic molecules is described as a 1st order reaction in most photocatalysis investigations using the following equation [57]:

(7) $$v=-\frac{dC}{dt}=k_{app}.C$$

*v*—the reaction rate (mg/L min); *C*—concentration of pollutant (mg/L); $k_{app}$:—the apparent rate constant (min^−1^).

The following equation is obtained by integrating Equation (7) [58]:

(8) $$ln\frac{C_{o}}{C}=k_{app}.t$$

where $C_{o}$ is the initial concentration of the pollutants. $k_{app}$ can be determined from the slope of the curve drawn between in $\frac{C_{o}}{C}$ and light irradiation time.

**Figure 9:**
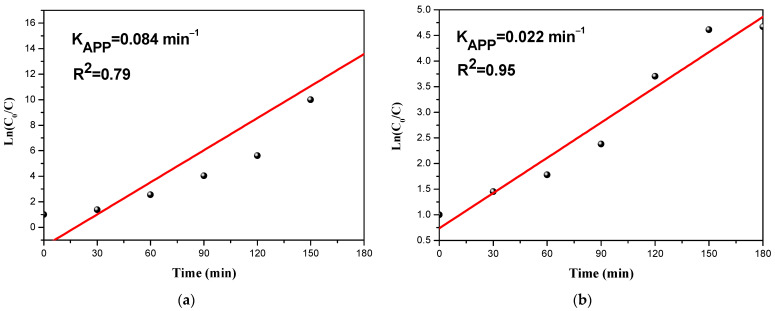
Pseudo-first-order kinetic plot (**a**) of the CFX degradation (**b**) of Cr(VI) reduction.

To check the order of the reaction, we plotted $\frac{C_{o}}{C}$ as a function of time for both pollutants (Figure 9). The obtaining of a straight line indicates that the kinetics is of the first order. Additionally, the first-order pseudo-model validation is confirmed by R^2^ values of the removal kinetics.

The apparent rate constant was calculated to be 0.084 min^−1^ and 0.022 min^−1^, respectively, for CFX, and Cr(VI). The rate constant for the organic pollutant degradation was found to be higher than that of inorganic compounds. This means that the degradation of organic pollutants was faster than the reduction of inorganic pollutants.

Table 2 summarizes a list of bibliographical references with the use of sillenites for photocatalytic application to remove both organic and inorganic pollutants. Our catalyst has exhibited unusual photocatalytic activity in both degradation and reduction compared to other sillenites used in earlier studies.

### 3.4. Proposed Mechanism of the Photocatalytic Removal

**Figure 10:**
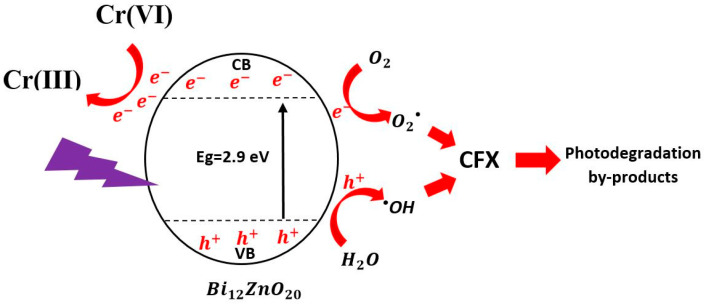
Possible mechanism of the photocatalytic removal of CFX and Cr(VI) using Bi_12_ZnO_20_.

According to previous studies dealing with photocatalytic elimination of organic or/and inorganic pollutants [35,61,62], the possible mechanism of the photocatalytic process can be summarized by the schematic in Figure 10.

When a light source (energy more than the bandgap) is exposed and exceeds the photocatalyst, electrons (e^−^) and holes (h^+^) occur in the conduction band (CB) and valance band (VB), respectively [1]. These pairs are responsible for eliminating organic or inorganic pollutants through reduction and oxidation reactions [26].

Starting with the oxidation reaction, which is responsible for the degradation of organic pollutants, in our case CFX, the holes (h^+^) in the valance band react and oxidize H_2_O molecules to produce hydroxyl radicals, ^•^OH, which are the most numerous and primary active species among the ROS in the oxidation of organic pollutants [1,13,63].

We used scavengers in our previous studies to focus on the contribution of reactive oxygenated species, and we validated that the radical most involved in the degradation process of organic compounds are OH° radicals [13].

On the other hand, the reduction reaction is responsible for eliminating the inorganic pollutant, in our case Cr(IV). The electrons (e^−^) in the conduction band reacted directly with Cr(VI) and reduced it to a lower valence state, Cr (III) [25,63,64]. This reduction reaction can minimize the risk of Cr(IV) by turning it into Cr (III) [1]. The e^−^ can also react and reduce oxygen molecules present in the solution, leading to the formation of superoxide radical O_2_^•^. These O_2_^•^ species can also participate in the degradation of organic pollutants through direct reaction with the pollutant but it is not an efficient [64] or indirect reaction as it undergoes a reaction that generates OH [1].

### 3.5. Catalyst Regeneration Study

**Figure 11:**
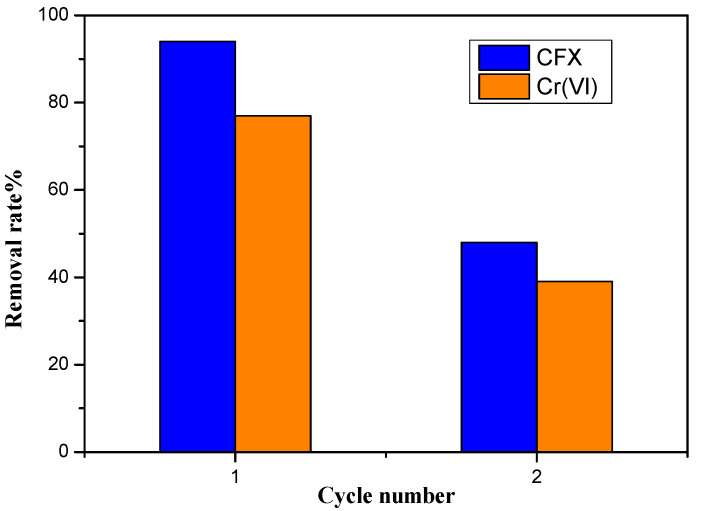
Photocatalytic efficiency of Bi_12_ZnO_20_ for two successive cycles.

The reuse and stability of a photocatalyst are critical proprieties for practical applications. BZO was tested in two successive series of photocatalytic reduction and degradation, for both Cr(VI) and CFX. After the first test, which was within 180 min of light irradiation, the catalyst was separated from the aqueous suspension by centrifugation, washed with HNO_3_ (1 M), ethanol, and then water, and then finally dried at 120 °C for 8 h. Then, the obtained catalyst was subjected to a second test with the same conditions. Figure 11 displays the performance of BZO over two successive photocatalytic applications. As can be observed, a decrease from the first to the second test for both degradation and reduction efficiency was recorded. This result can be explained by the effect of the catalyst’s longer electronic life (photosensitization), as it remains in the excitation state for a long time, which means a low recombination rate of the photogenerated electron–hole pairs (e^−^/h^+^) [65]. This finding is in line with the photoluminescence results. This decrease can be rectified by doping the catalyst with an ion, such as Ag, in order to speed up the electronic life [66]. This will be one of our outlooks for upcoming studies.

## 4. Conclusions

The sillenite crystals of Bi_12_ZnO_20_ were synthesized using the sol–gel method. XRD analysis confirmed the formation of a BZO cubic phase with a space group I23 and crystalline size (~59.46 nm). Raman analysis was used to verify the obtained sillenite phase. The morphology of this catalyst was investigated using SEM analysis. The optical properties showed its potential for photocatalytic applications in the UV region, and its bandgap of 2.9 eV allows UV radiation conversion. The photocatalytic performance of the BZO sillenite crystals was examined for degradation of cefixime (CFX) and the reduction of hexavalent chromium (Cr(VI)), as organic and inorganic compounds, respectively. The findings revealed that the BZO photocatalyst could lead to a 94.34% degradation of CFX and 77.19% reduction of Cr(VI) within 3 h of UV irradiation, which is very efficient compared to literature. According to these results, sillenite Bi_12_ZnO_20_ could be a promising catalyst for degradation and reduction applications, for treating both organic and inorganic pollutants in wastewaters.

## Figures and Tables

**Table 1:** Structural and lattice parameters.

**Phase**	Bi_12_ZnO_20_					
**Groupe Space**	I 2 3					
**a (Å)**	10.209611					
**Atoms**	Atom	x	y	z	Occupancy	Biso
	Zn	0.000000	0.000000	0.000000	1	0.21184404
	Bi	0.8251013	0.6822033	0.98639077	1	0.28883246
	O1	0.31147724	0.31147724	0.31147724	1	0.2218582
	O2	0.3605947	0.23639126	0.022656312	1	0.21213703
	O3	0.006910957	0.006910957	0.006910957	1	0.20685276
**V (Å^3^)**		1064.210594				
**D (nm)**		59.46				
**R Factors**	Rb Rexp Rwp Sig	24.588018 17.532957 30.810946 1.757316				

**Table 2:** Sillenites used for photocatalytic applications.

Sillenite	Organic Pollutants	Operating Conditions	Degradation Efficiency	Ref
Bi_12_ZnO_20_	Cefixime	Catalyst dosage: 1 g/L pH: 6, Reaction time: 3 h Initial concentration: 10 mg/L	94.34%	Present study
Bi_12_CoO_20_	Basic red 46	Catalyst dosage: 1 g/L pH: 6.4, Reaction time: 3 h Initial concentration: 15 mg/L	86%	[35]
Bi_12_ZnO_20_	Cefuroxime	Catalyst dosage: 1 g/L pH: 6, Reaction time: 4 h Initial concentration: 5 mg/L	80%	[36]
Bi_12_TiO_20_	Rhodamine B (RhB)	Catalyst dosage: 60 mg pH: 6, Reaction time: 210 min Initial concentration: 10 mg/L	81%	[32]
Bi_24_AlO_39_	Methyl Orange	Catalyst dosage: 6 g/L pH: 2, Reaction time: 2 h Initial concentration: 20 mg/l	100%	[59]
Bi_25_GaO_39_	Methylen blue	Catalyst dosage: 2 g/L, Reaction time: 60 min Initial concentration: 10 mg/L	89%	[30]
Bi_12_FeO_20_	Methylene blue and Congo red	Reaction time: 3.5 h Initial concentration: 3.5 mg/L and 10 mg/L	91.8% and 32.10%	[60]
**Sillenite**	**Inorganic pollutants**	**Operating conditions**	**Reduction efficiency**	**Ref**
Bi_12_ZnO_20_	Hexavalent chromium	Catalyst dosage: 1 g/L pH: 6, Reaction time: 3 h Initial concentration: 30 mg/L	77.19%	Present study
Bi_12_CoO_20_	Hexavalent chromium	Catalyst dosage: 1 g/L pH: 6.4, Reaction time: 3 h Initial concentration: 15 mg/L	67%	[35]
Bi_12_MnO_20_	Hexavalent chromium	Catalyst dosage: 2 g/L pH: 6, Reaction time: 4 h Initial concentration: 10 mg/L	80%	[29]

## Data Availability

Not applicable.
